# Syngas Production from Pyrolysis of Nine Composts Obtained from Nonhybrid and Hybrid Perennial Grasses

**DOI:** 10.1155/2014/723092

**Published:** 2014-07-01

**Authors:** Adéla Hlavsová, Agnieszka Corsaro, Helena Raclavská, Dagmar Juchelková, Hana Škrobánková, Jan Frydrych

**Affiliations:** ^1^ENET-Energy Units for Utilization of Non-Traditional Energy Sources, VŠB-Technical University of Ostrava, 17. listopadu 15/2172, 708 33 Ostrava-Poruba, Czech Republic; ^2^Institute of Geological Engineering, Faculty of Mining and Geology, VŠB-Technical University of Ostrava, 17. listopadu 15/2172, 708 33 Ostrava-Poruba, Czech Republic; ^3^Department of Energy, Faculty of Mechanical Engineering, VŠB-Technical University of Ostrava, 17. listopadu 15/2172, 708 33 Ostrava-Poruba, Czech Republic; ^4^OSEVA PRO s.r.o., Grass Research Institute, Rožnov-Zubří, Hamerská 698, 756 54 Zubří, Czech Republic

## Abstract

A pyrolysis of compost for the production of syngas with an explicit H_2_/CO = 2 or H_2_/CO = 3 was investigated in this study. The composts were obtained from nonhybrid (perennial) grasses (NHG) and hybrid (perennial) grasses (HG). Discrepancies in H_2_ evolution profiles were found between NHG and HG composts. In addition, positive correlations for NHG composts were obtained between (i) H_2_ yield and lignin content, (ii) H_2_ yield and potassium content, and (iii) CO yield and cellulose content. All composts resulted in H_2_/CO = 2 and five of the nine composts resulted in H_2_/CO = 3. Exceptionally large higher heating values (HHVs) of pyrolysis gas, very close to HHVs of feedstock, were obtained for composts made from mountain brome (MB, 16.23 MJ/kg), hybrid Becva (FB, 16.45 MJ/kg), and tall fescue (TF, 17.43 MJ/kg). The MB and FB composts resulted in the highest syngas formation with H_2_/CO = 2, whereas TF compost resulted in the highest syngas formation with H_2_/CO = 3.

## 1. Introduction

The pressing demands for greater generation of energy at a lower cost, associated with a diminution of greenhouse gases (GHG) emission, have compelled researchers to expand their search for an energy source outside conventional and primary energy sources, such as fossil fuels. This pursuit is facilitated by the utilization of renewable energy sources and based on Directive 2001/77/EC of the European Parliament that must consist of 13% of the total energy consumption by 2020 in the Czech Republic [1]. Biomass, specifically energy crops, is of particular interest among these renewable energy resources. It has been predicted that by 2050 energy crops will have the potential to supply around 200–400 EJ/year at a competitive cost [2], and up to 161 EJ/year of the 200–400 EJ/year range will come from projected surplus cropland and grassing areas [3]. The competitive costs are based upon the incentives made available through the scheme for energy crops according to the Article 88 of Regulation (EC) No. 1782/2003 [4]. The preference of energy crops over other types of biomass for energy generation is due to their (i) higher productivity, (ii) lower investment cost, (iii) low environmental maintenance, (iv) short time between plantation and harvesting, and (v) high energy values [5–7]. Another reason for which energy crops, in particular grasses, are being considered as a source of energy is the overproduction of grass and/or hay from permanent grasslands. This overproduction is a result of a diminution of livestock. According to the Czech Statistical Office, the land used for agriculture reached 959,131 ha with harvest of 3.22 t/ha in 2012 [8]. Comparatively, livestock numbers decreased since 1990 by 60.78% for cattle and 66.74% for pigs reaching 1375 cattle and 1593 pigs in 2012 [9]. Therefore, it is necessary to find an appropriate technology to manage and utilize the excess of grass.

The number of technologies available to convert biomass to energy has developed greatly in recent years and they are focused in general on production of synthesis gas (syngas) [10, 11]. Syngas which is a mixture of hydrogen (H_2_) and carbon monoxide (CO) can then be directly converted to energy through combustion or to a variety of fuels: (i) liquid hydrocarbons from methanol, (ii) liquid hydrocarbons through Fischer-Tropsch synthesis, and (iii) synthetic natural gas (SNG) [11, 12]. The selective conversion of syngas to liquid hydrocarbons or SNG requires, however, specific ratios of H_2_ to CO in the amount of 2 or 3, respectively [12]. Obtaining those explicit values is not a straightforward task as the yields of produced noncondensable gases depend on several factors such as raw material composition and operating conditions of the converting process [13–15].

Two methods in particular have been reported in the literature that convert biomass to syngas, namely, gasification and pyrolysis. Gasification is a thermochemical method which converts a variety of biomass in an oxygen environment. Typical reactions involved in any gasification process are the reactions using oxygen (O_2_) (combustion) represented by (1) and (2), the reverse Boudouard reaction represented by (3), the water-gas reaction represented by (4), and the water-gas shift (WGS) reaction represented by (5) [16, 17]:

(1) Partial  oxidation:2C+O2⟶2CO ΔHr0=−222 kJ/mol

(2) Oxidation  of  carbon:C+O2⟶CO2 ΔHr0=−394 kJ/mol

(3) Revers  Boudouard  reaction:C+CO2⟶2CO ΔHr0=173 kJ/mol

(4) Water-gas  reaction:C+H2O⟶CO+H2 ΔHr0=131 kJ/mol

(5) Water-gas  shift  reaction:CO+H2O⟶CO2+H2 ΔHr0=−41 kJ/mol

As a consequence of these reactions, a wide range of H_2_/CO ratios (0.45–2) are obtained [18]. Pyrolysis on the other hand is a process in which biomass undergoes thermal degradation in an oxygen-free atmosphere. The final products are pyrolysis solid, liquid, and gas containing mainly CO, carbon dioxide (CO_2_), H_2_, and lower hydrocarbons (C_1_–C_3_). The remaining reactions involved in the process apart from previously mentioned reactions (3), (4), and (5) are the following [19]:

(6) Steam  reforming  of  methane:CH4+H2O⟶CO+3H2 ΔHr0=206 kJ/mol

(7) Steam  reforming  of  tar:CnHmOp+(2n−p)H2O⟶nCO2+(1/2m+2n−p)H2 ΔHr0≥0 kJ/mol

(8) Thermal  cracking:CnHm⟶Cn−xOm−y+H2+CH4+C ΔHr0≥0 kJ/mol

(9) Methanation:C+2H2⟶CH4 ΔHr0=−75 kJ/mol

The obtained H_2_/CO ratios are dependent on pyrolysis temperature and increase as the latter increases [15, 20–22]. Their values are, however, somewhat lower (i.e., 0.1–1.42 in the 500–1000°C range) when compared to the values obtained from biomass gasification [15, 20, 22]. In addition, in order to make syngas suitable for commercial application (i.e., reduce economic investment and improve its quality (obtain an appropriate H_2_/CO ratio,* ipso facto* increase H_2_ formation)), the obtained gas mixture needs to be cleaned and processed [18, 23]. These requirements can be avoided or minimized by pretreatments of biomass raw material such as composting [10, 24].

Composting is a naturally occurring, biological decomposition process in which bacteria, fungi, and other microorganisms break down the organic matter into a more stable form called compost [10, 24, 25]. The process occurs in two stages. The first stage (i.e., organic matter degradation) results in the formation of CO_2_, NH_3_, H_2_O, saccharides, and humic substances (fulvic and humic acids) with emission of heat [23, 26]. The second stage involves the maturation and stabilization of formed material [26]. As a result of both stages, the newly formed organic matter has a different chemical composition,* ipso facto* thermal behavior [10, 23, 24, 27]. Composting reduces the content of two of the major biomass components, namely, cellulose and hemicellulose, while increasing the content of lignin [10, 23, 24]. These changes are of particular importance, since lignin is the component responsible for the highest H_2_ and CH_4_ formation, hemicellulose is responsible for the highest CO_2_ emission, and cellulose is responsible for the highest CO release [28–31]. Yang et al. [29] examined pyrolysis characteristics of lignin, cellulose, and hemicellulose and concluded that the main source of H_2_ release was lignin. Lignin resulted in four times more emission of H_2_ than cellulose and three times more than hemicellulose [29]. Similar results were obtained by Barneto et al. [31] who extended the investigation of H_2_ origin and reported that although most of H_2_ is emitted from thermal degradation of lignin, three times more H_2_ is released from charring than from volatilization of lignin. In addition, hemicellulose is the least stable from the three biochemical components and, therefore, reacts at the lowest temperatures, followed by cellulose and lignin [32]. Consequently, the changes in the chemical composition due to composting result in the changes in pyrolysis gaseous products. That is, a mixture of H_2_ and CO becomes the primary product, not a mixture of CO_2_ and CO which are the major products of biomass pyrolysis [21, 28]. For this reason also, the composts obtained from perennial grasses rather than grasses alone are considered as a feedstock for pyrolysis experiments in this research.

The purpose of this study was to compare the composition, yields, and evolution of gaseous products from pyrolysis of nine composts. The examined composts were obtained from two types of perennial grasses: nonhybrid and hybrid. The main objective was selective formation of syngas with an explicit H_2_/CO ratio in the amount of 2 : 1 or 3 : 1.

## 2. Materials and Methods

### 2.1. Materials

Nine composts made from perennial grasses (six nonhybrid grasses (NHG) and three hybrid grasses (HG)) were investigated in this study. The grass crops were obtained from OSEVA PRO s.r.o., Grass Research Institute, Rožnov-Zubří, CZ. The names and abbreviation of the composts examined are displayed in Table 1.

### 2.2. Composting

The composting experiments were carried out at the Institute of Geological Engineering, VŠB-Technical University of Ostrava (VŠB-TU Ostrava), CZ. The nine perennial grasses were finely chopped (<2 cm) and mechanically mixed with sawdust and soil in the ratio of 4 : 2 : 1 in order to obtain the appropriate C/N ratio. The composting of each blend (perennial grass, sawdust, and soil) was conducted in a microcomposter (NM125, NatureMill) for 10 days, whereas the maturation of composts was carried out for 14 days.

### 2.3. Chemical Characterization of Composts

All nine composts were subjected to proximate and ultimate analyses. The following standard test methods were applied: CSN EN 15402 (volatile matter), CSN EN 15403 (ash), CSN EN 15104 (carbon (C), nitrogen (N), and hydrogen (H)), and CSN EN 15400 (higher heating value (HHV)). The summarized results are presented in Table 2. The biochemical components were determined according to the CSN EN ISO 13906 standard test method (lignin) and the method described by Kačík and Solár [33] (cellulose and hemicellulose). Humic acids (HA) and fulvic acids (FA) were extracted from composts according to the method described by Swift [34]. In addition, analysis of water-soluble alkali was conducted according to the EN 15105 standard test method. The summarized results are shown in Table 3.

### 2.4. Pyrolysis Experiments

**Figure 1:**
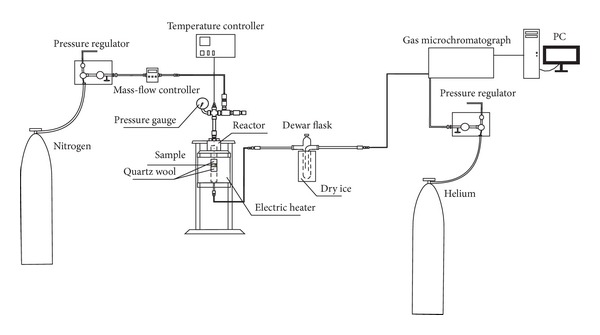
Experimental setup.

The pyrolysis experiments were conducted in a stainless steel fixed bed reactor equipped with an electric heater (Parr). The temperature of the heater was controlled by a temperature controller (Parr, 4836 controller), while the reaction temperature was monitored by a K-type thermocouple. The experiments were carried out in N_2_ atmosphere and the flow of gas was controlled by a mass- flow controller (SIERRA C100 Serie, Smart-Trak). The experimental setup is presented in Figure 1. For all pyrolysis experiments, 0.5 g of compost was loaded into the reactor and heated to a final temperature of 700°C. The flow of N_2_ was maintained at 20 smL/min for all experiments. The experiments were considered completed when N_2_ was the only gas detected by online gas chromatography (GC).

### 2.5. Analysis of Gas Product

The noncondensable pyrolysis product was analyzed by online 2-channel GC (Agilent 3000) equipped with thermal conductivity detectors. The channels were equipped with the following columns: Molsieve for separation of H_2_, N_2_, CO, and CH_4_ and PLOT U for separation of CO_2_, C_2_, and C_3_.

### 2.6. Statistical Analysis

The relationships between components of chemical analyses and pyrolysis gaseous products yields were tested by bivariate correlation analysis, specifically Pearson's correlations. SPSS 17 statistical software was applied.

## 3. Results and Discussion

### 3.1. Gas Yield and Evolution

**Figure 2:**
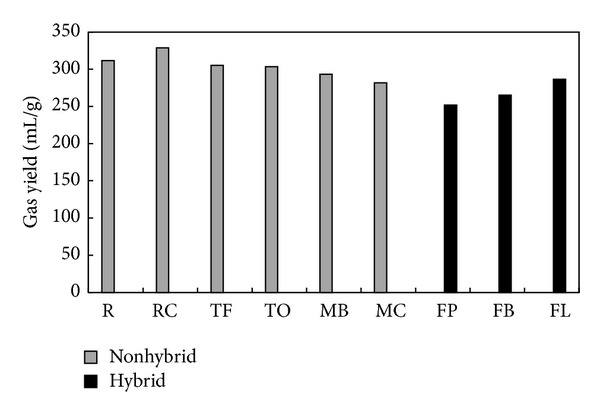
Total gas yield of composts.

The yield of gaseous products obtained from pyrolysis of NHG and HG composts referred to as a gram of compost used is presented in Figure 2. The highest yield of pyrolysis gaseous products among NHG composts (328.81 mL/g, also the highest yield among all composts pyrolyzed) was obtained for RC compost, whereas the lowest yield of pyrolysis gaseous products (281.74 mL/g) among NHG composts was obtained for MC compost. The highest pyrolysis gas yield among HG composts was obtained for FL compost (286.41 mL/g), whereas the lowest pyrolysis gas yield was obtained for FP compost (251.77 mL/g) which also exhibited the lowest gas yield among all composts examined. The yield of pyrolysis gas decreased in the following order: RC > R > TF > TO > MB > MC for composts made from nonhybrid grasses and FL > FB > FP for composts made from hybrid grasses.

**Figure 3:**
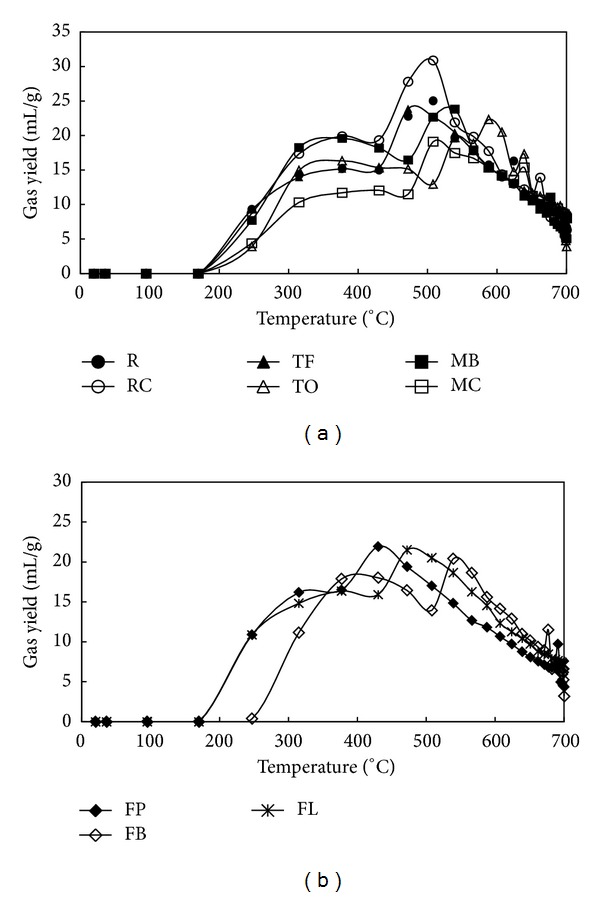
Evolution of gas released during pyrolysis of composts: (a) NHG and (b) HG as a function of temperature.

Figure 3 shows the evolution profiles of released pyrolysis gas as a function of temperature.  Figure 3(a) presents the evolution profiles of gas released during pyrolysis of NHG composts, while Figure 3(b) shows the evolution profiles of gas released during pyrolysis of HG composts. The emission of noncondensable pyrolysis gases at temperature below 500°C is related in majority to degradation of the biochemical structures of compost as well as humic substances formed during the composting process [21, 23, 35, 36], whereas the release of gases at temperature >500°C is likely associated with secondary reactions of char formed from biochemical components or humic substances [21, 23, 28, 35, 36]. In general, pyrolysis gases began to release at 170°C for all compost samples examined which is equivalent to the beginning of thermal degradation of hemicellulosic fraction [23]. The distinction was only observed for the pyrolysis experiment conducted on compost made from hybrid grass (FB) for which a beginning of gas evolution at 247°C was observed. Apart from TO compost which exhibited a three-stage evolution profile (three peaks were observed), all composts investigated resulted in two-stage evolution profiles (two peaks were observed). The composts made from nonhybrid grasses exhibited the maximum of the first peak at temperature range of 315–430°C which is a typical temperature range of thermal degradation of cellulose fraction [29, 30, 37]. The maximum of the second peak was observed at 472°C for TF compost, 508°C for R, RC, and MC composts, and 539°C for TO and MB composts and can be mainly attributed to thermal degradation of lignin and secondary reactions of chars and liquids [23, 29, 30, 37]. The third peak observed for TO compost was detected at 588°C and is also likely due to thermal degradation of lignin and secondary reactions of chars and liquids [23, 29, 30, 37]. As previously mentioned, composts made from hybrid grasses resulted in two-stage gas evolution profiles as well, although more noticeable shifts in the peaks maximum were observed. Specifically, a shift from maximum at 315°C to maximum at 377°C was observed for FP, FB, and FL composts, respectively, and a shift from maximum at 430°C to maximum at 472°C and to maximum at 539°C for the second peak was observed for FP, FL, and FB composts, correspondingly. The change of peaks maximum noted for the FB composts is likely due to the delay of starting point of pyrolysis gas release.

### 3.2. Pyrolysis Gas Composition

**Figure 4:**
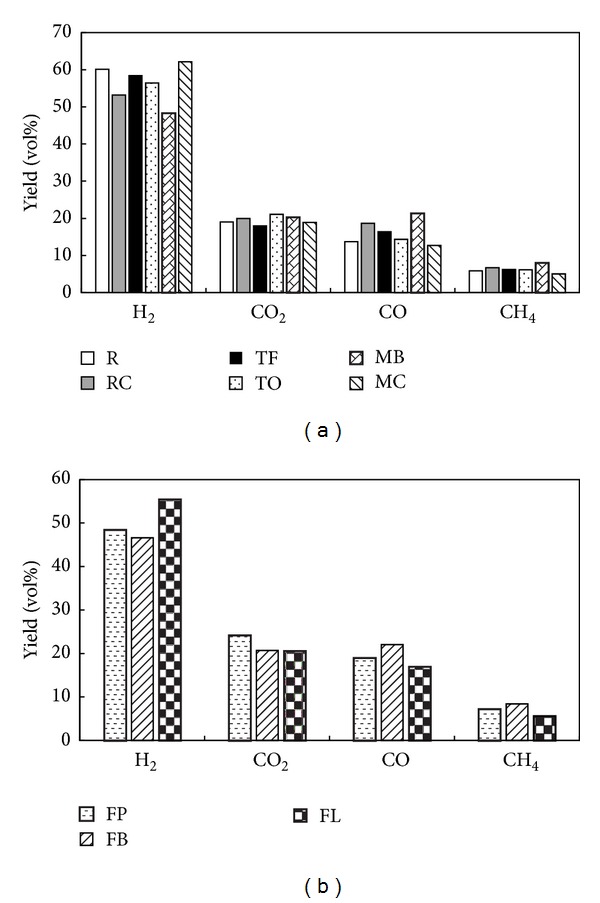
Yields of individual gaseous products from pyrolysis of (a) NHG composts and (b) HG composts at 700°C and N_2_ free-vol%.

The yields of major pyrolysis gaseous products (H_2_, CO_2_, CO, and CH_4_) from grass composts are presented in Figure 4. Other products such as short-chain hydrocarbons (i.e., C_2_ and C_3_) were also detected but in sizably lower amounts (less than 1 vol%) and will not be discussed.  Figure 4(a) shows yields of NHG composts gaseous products, whereas Figure 4(b) shows yields of HG composts gaseous products. The yields were calculated at 700°C (after 112 min) and at N_2_ free-vol%. Among NHG composts, MC compost resulted in the highest production of H_2_ (62.17 vol%), the lowest formation of CO (12.74 vol%) and CH_4_ (5.10 vol%), and the second lowest formation of CO_2_ (18.93 vol%). The lowest yield of H_2_ (48.32 vol%) was observed for MB compost and as expected it also resulted in the highest CO (21.34 vol%) and CH_4_ (8.01 vol%) formation and a moderately high formation of CO_2_ (20.30 vol%). The majority of these observations are directly related to the biochemical composition of examined composts and the contents of water-soluble alkali. Specifically, lignin, cellulose, and potassium (K) contents were found to be associated with H_2_ as well as CO and CH_4_ formation. A positive correlation was observed between H_2_ yield and lignin (*R* = 0.916, *P* < 0.05), and stronger negative correlations were observed between CO yield and lignin (*R* = −0.974, *P* < 0.01) and between CH_4_ yield and lignin (*R* = −0.929, *P* < 0.01). The relationship between H_2_ and lignin is consistent with the results obtained by Barneto et al. [10] who examined the effect of* Leucaena* and tagasaste composts on the production of volatiles from pyrolysis and reported 75 wt% production of H_2_ from lignin. A positive correlation was also observed between H_2_ yield and K content (*R* = 0.750, *P* < 0.1) and negative correlations were obtained between CO and CH_4_ yields and K content (*R* = −0.901, *P* < 0.05 and *R* = −0.742, *P* < 0.1, correspondingly). Negative relationships between K content and CO and CH_4_ were likewise observed by Couhert et al. [38] who reported that mineral matter can influence pyrolysis reactions occurring inside the component's particle and decrease the formation of aforementioned gases. As previously mentioned, these correlations can also be explained by the occurrence of char gasification reactions ((4) and (5)) which are likely to be a result of combination of lignin and K contents. A higher lignin content is associated with a higher K content (i.e., Pearson's correlation coefficient between lignin and K content was 0.832, *P* < 0.05) [28, 39]. Potassium, on the other hand, is known to be an effective catalyst for char gasification [20, 24, 39]. Both MC and MB composts have shown the highest and the lowest lignin and K contents which would explain their H_2_ and CO yields, the highest and lowest for MC compost, and the opposite for MB compost, respectively. As previously noted, the formation of H_2_ in majority from charring reactions was also confirmed by Barneto et al. [10]. Opposite correlations to those observed between lignin content and CO, CH_4_, and H_2_ yields were found for cellulose. That is, a negative correlation was calculated between H_2_ yield and cellulose content (*R* = −0.860, *P* < 0.05), and positive correlations were found between CO yield and cellulose content (*R* = 0.952, *P* < 0.01) and between CH_4_ yield and cellulose content (*R* = 0.876, *P* < 0.05). The strong positive relationship between CO yield and cellulose content is directly related to higher content of carbonyl groups in cellulose, which is consistent with results obtained by Qu et al. [28] and Yang et al. [29]. The exception to the observed correlations was observed for CO_2_ yield which was found to be unrelated to either biochemical composition or water-soluble alkali. Instead, a weaker and marginally significant correlation to one of the components of proximate analysis, moisture, was observed (*R* = 0.780, *P* < 0.1), which is a further confirmation of presence of water-gas shift reaction.

The individual products yields trends observed for NHG composts were also observed for two out of three HG composts, namely, FP and FB (Figure 4(b)). The remaining compost, FL, resulted in the highest yield of H_2_ (55.43 vol%) and the lowest yields of CO_2_ (20.58 vol%), CO (16.95 vol%), and CH_4_ (5.60 vol%). The analogy of NHG composts cannot be, however, applied to these samples as the correlation between lignin and K contents was in the opposite direction (*R* = −0.983) with a value which fell just shy of the statistical significance threshold (*P* = 0.127). A negative correlation between lignin and K was also reported by Fahmi et al. [40] who investigated the effect of alkali metals on pyrolysis of* Lolium* and* Festuca* independently. For this reason, the NHG and HG composts samples were also separated in this study when examining the possible relationships between gaseous product yields and composts composition. In addition, the correlations between individual products' (H_2_, CO, and CH_4_) yields and lignin content were no longer applicable and insignificant due to the small number of observations. The observed changes may, however, suggest that as much as both lignin and K contents affect the H_2_ formation during pyrolysis of NHG composts, in the case of HG composts, it may be K content that has the greatest influence on H_2_ production.

**Figure 5:**
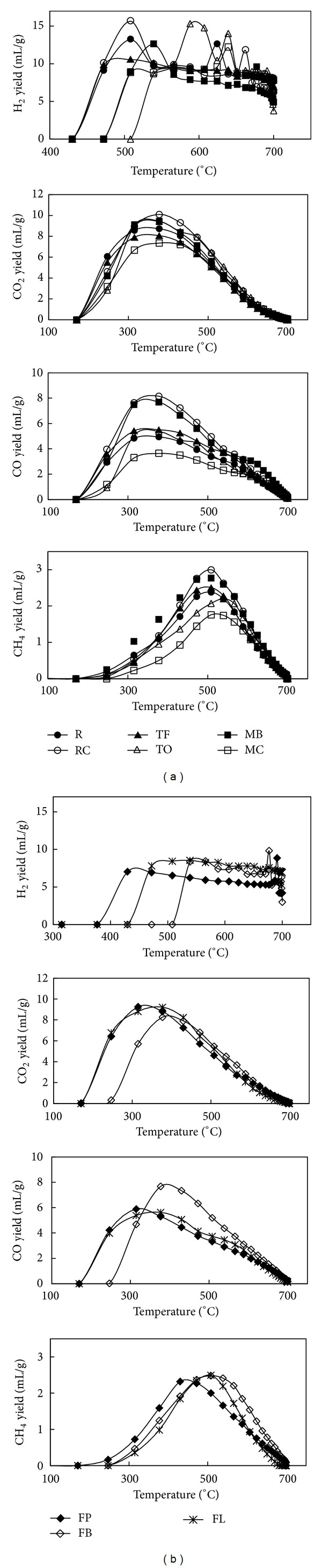
Evolution profiles of individual gaseous products from pyrolysis of (a) NHG composts and (b) HG composts.

Figure 5 presents the evolution profiles of pyrolysis gaseous products of NHG composts (Figure 5(a)) and HG composts (Figure 5(b)) as a function of temperature. The products released at temperature below 450°C consisted mainly of CO_2_, CO, and CH_4_ which is consistent with the prior literature [21]. A further increase of pyrolysis temperature changed the emission of pyrolysis gases as the yield of CO and CO_2_ began to decrease in expense of greater H_2_ and CH_4_ formation. The greatest discrepancies in the emission profiles of primary noncondensable gases were observed for H_2_ profiles. The differences occurred not only in the gas release temperature but in the shape of evolution profiles as well. The emission of H_2_ during pyrolysis of NHG composts began at 430°C (R, RC, and TF composts), 472°C (MC and MB composts), and 508°C (TO compost), whereas the release of H_2_ during pyrolysis of HG composts began at 377°C (FP compost), 430°C (FL compost), and 508°C (FB compost). The majority of NHG composts (R, RC, TO, and MB) exhibited double-peak profiles with some shift of both the first peak maximum (observed in the 508–588°C range) and the second peak maximum (observed in the 624–677°C range). The maximum of the first peak is the highest and is attributed to cracking of C–H bonds of lignin and cellulose and its shift is a consequence of a change of released temperature [29], whereas the second peak is smaller and is associated with pyrolytic reactions of lignin due to its higher content of aromatic ring (i.e., cracking and deformation of C=C and C-H bonds) and charring reactions [29, 31]. The observed changes are also a further confirmation of the fact that the formation of H_2_ at temperature >400°C is mostly contributed by pyrolysis of biochemical components, whereas the release of H_2_ at temperature >500°C is mainly attributed to thermal degradation of lignin and the occurrence of charring reactions [21, 35, 36]. As aforementioned, these reactions are more pronounced in the energy grasses than in other biomass type materials (i.e., wood) due to a greater amount of alkali metals responsible for catalyzing these types of reactions [35, 36]. The remaining NHG composts, MC and TF, resulted in single-peak or no-peak evolution profiles, respectively. Similar H_2_ profiles were exhibited by HG compost; specifically, a single-peak profile was obtained for FP and FB composts, whereas a no-peak profile was obtained for FL compost. The resemblance between HG composts corresponds well with the profile obtained for TF compost since HG composts are a cross between* Festuca* (FT) and* Lolium*. The overall yield of formed H_2_ in these samples is also related to a combination of both pyrolytic reactions of biochemical compounds and charring reactions.

No significant discrepancies between NHG and HG composts were observed in the emission profiles of the remaining gaseous products. All samples displayed wide single-peak profiles and, in general, a starting point of emission at 170°C. The shift of a starting point of emission to 247°C was only observed for FB compost for CO and CO_2_ evolution profiles. The majority of CO_2_ release took place in the temperature ranges of 250–450°C for FP and FL (hybrid grasses) composts, and 300–500°C for NHG and FB (hybrid grass) composts. This corresponds well with CO_2_ release from all biochemical components through cracking and reforming of carboxyl groups [28, 29, 41] and is in agreement with calculated Pearson's correlations (i.e., no single statistically significant relationship towards one particular biochemical component was observed). A reduction of CO_2_ emission at temperature >500°C is likely due to secondary reactions of volatiles as temperature at this point has a limited influence [21, 42]. A minor difference between both types of composts was observed in the emission of CO. That is, a single evolution profile with a release of majority of the product in 300–500°C range was obtained for RC, MB (nonhybrid grasses), and FB (hybrid grass) composts. The remaining samples exhibited a wider but shorter CO peak at temperature ranges of 300–500°C and 250–500°C with a break of possible second peak at 500–640°C range for R, TF, TO, and MC (nonhybrid grasses) and FP and FL (hybrid grasses) composts, correspondingly. The CO emission is mainly attributed to cracking of carbonyl and carboxyl groups from cellulose [28, 29, 41]. The most constant evolution profile was obtained for CH_4_ as its emission focused mainly at a temperature range of 450–550°C and was attributed to cracking of methoxyl groups [28, 29, 41].

### 3.3. Syngas Production

**Figure 6:**
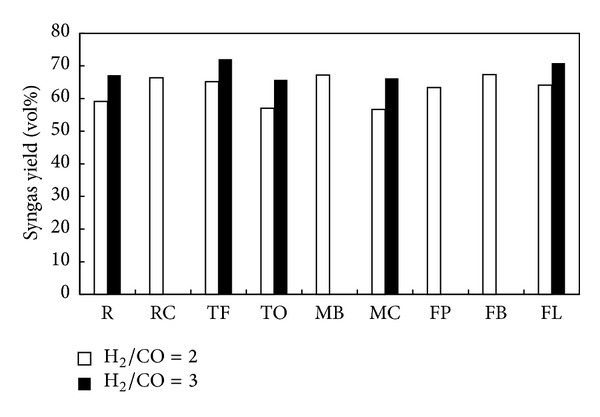
Total yield of syngas at H_2_/CO = 2 and H_2_/CO = 3.

The high variability of CO and H_2_ yields led to gas mixtures with equally high variability of H_2_/CO ratios.  Figure 6 presents total yield of formed syngas with respect to the particular H_2_/CO ratio, specifically, the total yield of syngas produced with H_2_/CO = 2 used in Fischer-Tropsch and methanol syntheses and with H_2_/CO = 3 used for synthetic natural gas production. All compost samples investigated resulted in syngas formation with H_2_/CO = 2. However, only five composts resulted in the production of syngas with H_2_/CO = 3 (i.e., R, TF, TO, and MC composts obtained from nonhybrid grasses and FL compost from hybrid grass). The highest amount of syngas with H_2_/CO = 2 was obtained for MB (nonhybrid grass) and FB (hybrid grass) composts, 67.23 vol% and 67.38 vol%, respectively. These two composts also resulted in the lowest H_2_ and the highest CO yields, correspondingly, whereas the highest amount of syngas with H_2_/CO = 3 (72.10 vol%) was obtained from pyrolysis of TF (nonhybrid grass) compost.

**Figure 7:**
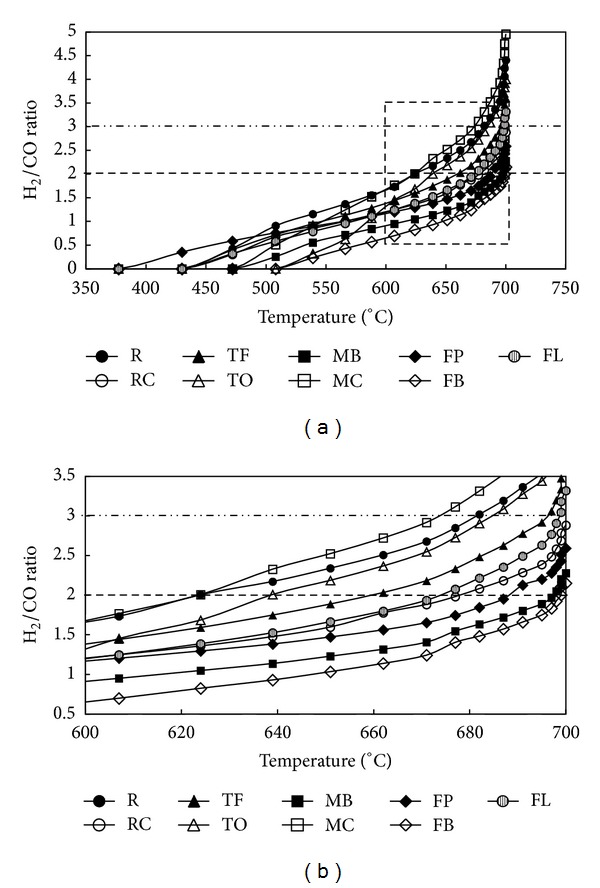
H_2_/CO ratio as a function of temperature.

The change of H_2_/CO ratio with pyrolysis temperature is shown in Figure 7. In general, apart from two samples, TO and MC composts, all composts displayed a gradual increase of H_2_/CO ratio with a pyrolysis temperature increase up to 650°C. A further increase of pyrolysis temperature resulted in a steep H_2_/CO ratio increase which corresponds well with obtained CO evolution profiles and indicates mostly H_2_ generation. The TO and MC composts exhibited a more abrupt increase of H_2_/CO ratio with a pyrolysis temperature increase, which can indicate a higher rate of charring and cracking reactions for these particular samples. It was not a surprise that in regard to the specific value of H_2_/CO ratio, these two composts reached this value at the lowest temperature. That is, a H_2_/CO ratio = 2 was obtained at 624 and 639°C, whereas a H_2_/CO ratio = 3 was obtained at 673 and 684°C, for MC and TO composts, respectively. Among samples for which H_2_/CO ratios increased gradually, only one sample in particular reached the required H_2_/CO ratio at a similar temperature range. Specifically, R composts resulted in H_2_/CO ratio = 2 at 624°C and in H_2_/CO ratio = 3 at 682°C. The remaining samples reached the essential H_2_/CO ratio in higher temperature ranges of 660–700°C and 695–700°C for H_2_/CO ratio = 2 and H_2_/CO ratio = 3, correspondingly. The temperature necessary to obtain the specific H_2_/CO ratios increased, therefore, in the following composts type order: (i) H_2_/CO = 2: R, MC < TO < TF < FL < RC < FP < MB < FB and (ii) H_2_/CO = 3: MC < R < TO < TF < FL.

### 3.4. Pyrolysis Gas HHV

**Figure 8:**
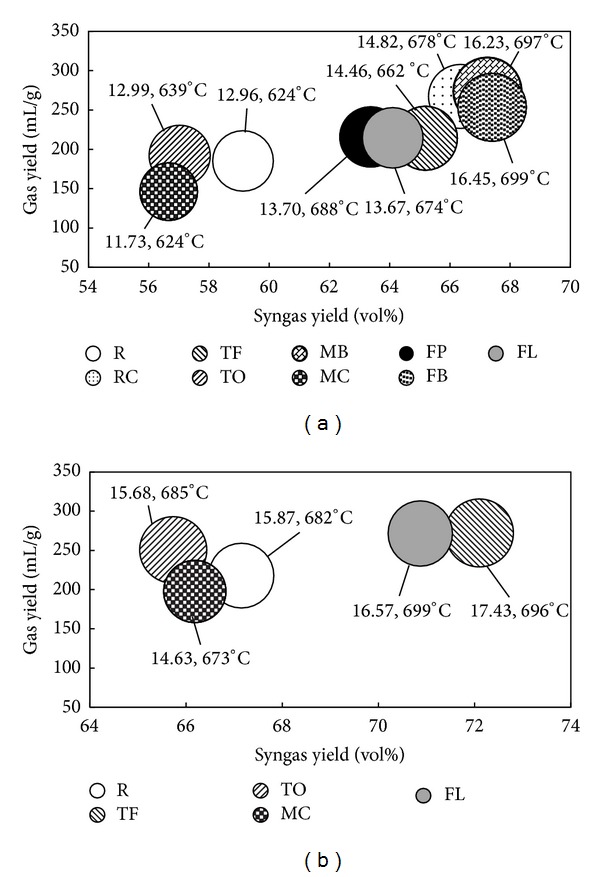
HHVs of pyrolysis gas with respect to its yield and syngas yield: (a) H_2_/CO = 2 and (b) H_2_/CO = 3.

The HHVs of pyrolysis gas with respect to its total yield and syngas yield are displayed in Figure 8. The size of the bubble represents the HHV expressed in MJ/kg_gas_ obtained at a specific pyrolysis temperature at which an explicit H_2_/CO ratio was reached.  Figure 8(a) shows the HHVs of pyrolysis gas obtained at H_2_/CO = 2, and Figure 8(b) shows the HHVs of pyrolysis gas obtained at H_2_/CO = 3. The HHV was directly associated with pyrolysis temperature; that is, as pyrolysis temperature increased, the HHV increased as well. As a consequence, the optimal HHV was reached at the highest syngas yield, which is consistent with the prior literature [21, 43, 44], but not at the highest gas yield. For example, the highest HHV at H_2_/CO = 2 in the amount of 16.23 and 16.45 MJ/kg_gas_ was obtained for MB (nonhybrid grass) and FB (hybrid grass) composts, respectively, which also resulted in the highest syngas formation; however, they exhibited one of the lowest total gas yields, whereas the highest HHV at H_2_/CO = 3 was obtained for TF (nonhybrid grass) and FL (hybrid grass) composts, 16.57 and 17.43 MJ/kg_gas_, correspondingly, which corresponded to the highest syngas yield and third highest total gas yield for TF compost and the highest total gas yield and second highest syngas yield for FL compost. It is important to note that these values are only marginally lower than the HHVs obtained for raw materials (i.e., by 5.69%, 9.17%, 4.22%, and 4.28% for MB, FB, TF, and FL composts, resp.) and are comparable to those obtained from pyrolysis of wood or coir pith [39, 45]. The HHVs of pyrolysis gas reported in the literature are significantly lower (4–12 MJ/kg_gas_) and are given mainly for products obtained from pyrolysis of grasses rather than grass composts [12, 42, 43, 46]. The significant increase of observed pyrolysis gases HHV is likely due to (i) composting process which results in lignin enriched material,* ipso facto* greater H_2_ formation, and (ii) sawdust addition to the composting process [47].

## 4. Conclusions

The syngas generation from pyrolysis of nine composts was investigated in this study. Composts were divided into two groups: composts obtained from nonhybrid perennial grasses and composts obtained from hybrid perennial grasses. The pyrolysis experiments were conducted in a fixed bed reactor to a final temperature of 700°C. Apart from compost obtained from tall oatgrass which exhibited the evolution gas profile with three peaks, all the remaining materials displayed two-peak evolution profiles indicating formation of gases based on two main processes: (i) thermal decomposition of biochemical components and (ii) secondary reactions of char. A distinction between NHG and HG composts was observed for the evolution profiles of individual gaseous products, in particular H_2_. HG composts resulted in no-peak evolution profiles, whereas NHG composts displayed two-peak and one-peak distributions. A no-peak distribution was only observed for one of the NHG composts made from tall fescue which belongs to the same genus as hybrid grasses. The remaining gaseous products (CO, CO_2_, and CH_4_) did not result in significant changes in emission profiles. It was found that all examined composts resulted in H_2_/CO necessary for future utilization in the amount of 2 and only five of the studied composts resulted in H_2_/CO = 3. A close relationship was established between H_2_, CO, and syngas yields, HHV of pyrolysis gas, and temperature required to obtain the essential H_2_/CO ratio. Specifically, composts obtaining the highest H_2_ yield resulted in the lowest CO yield, following the lowest syngas yield, HHV, and temperature required to obtain the specific H_2_/CO ratio, whereas composts obtaining the lowest H_2_ yield resulted in the highest CO yield, following the highest syngas yield, HHV, and temperature required to obtain the essential H_2_/CO ratio. It was also found that the formation of H_2_ was significantly correlated with lignin and K contents for nonhybrid grass. That is, strong positive correlations were obtained between (i) lignin and K contents, (ii) lignin content and H_2_ yield, and (iii) K content and H_2_ yield. Comparatively, a negative correlation (that just barely fell outside statistical significance) was observed for HG composts between lignin and K contents. The remaining correlations (i.e. between lignin content and H_2_ yield and between K content and H_2_ yield) were not statistically significant. Remarkable results were also obtained for HHV of pyrolysis gas. Specifically, values close to the HHVs of raw materials, 16.23, 17.43, and 16.45 MJ/kg, were calculated for composts obtained from mountain brome, tall fescue, and festulolium Becva composts, respectively. The increase was attributed to the composting process (i.e., an increase of lignin content) and sawdust addition. Finally, the composts made from mountain brome grass and hybrid Becva were recognized as the optimal materials for fuel/energy generation due to their (i) highest syngas formation and (ii) highest HHV.

## Figures and Tables

**Table 1:** Names and abbreviations of composts.

Grass type	Grass name	Compost abbreviation
Nonhybrid	Redtop-Rožnovský (*Agrostis gigantea *Roth)	R
	Reed canary grass-Chrastava (*Phalaris arundinacea* L.)	RC
	Tall fescue-Kora (*Festuca arundinacea* Schreb.)	TF
	Tall oat grass-Rožnovský (*Arrhenatherum elatius* L.)	TO
	Mountain brome-Tacit (*Bromus marginatus* Nees ex Steud.)	MB
	Mixture of clover (*Trifolium pratense*)	MC

Hybrid	Festulolium Perun	FP
	Festulolium Becva	FB
	Festulolium Lofa	FL

**Table 2:** Proximate and ultimate analyses of composts.

Compost	Ultimate analysis (wt%)^a,b^				Proximate analysis (wt%)				HHV (MJ/kg)
	C	N	H	O^c^	Moisture^d^	Volatile matter^a^	Ash^a^	Fixed carbon^a,c^	
R	47.24	0.91	6.6	45.25	4.54	74.88	09.83	15.29	17.45
RC	46.43	0.49	6.88	46.2	6.29	76.02	07.6	16.38	17.61
TF	46.39	0.56	7.09	45.97	4.38	74.17	10.3	15.53	17.3
TO	48.84	1.11	6.7	43.34	5.66	72.3	12.72	14.98	17.53
MB	47.9	0.59	7.14	44.37	5.87	73.1	11.42	15.48	17.21
MC	44.43	0.96	5.93	48.68	5.23	71.98	13.3	14.72	16.69
FP	46.5	0.83	6.6	46.07	6.12	73.29	10.52	16.19	18.29
FB	44.64	0.64	6.42	48.3	5.5	75.73	07.95	16.32	18.11
FL	48.11	0.77	6.52	44.6	5.68	74.26	08.94	16.8	18.21

^a^Dry basis.

^b^Ash free.

^c^Calculated by difference.

^d^As received.

**Table 3:** Biochemical components, humic to fulvic acids ratio, and water-soluble alkali contents of composts.

Compost	Lignin (wt%)	Cellulose (wt%)	Hemicellulose (wt%)	HA/FA	Na (mg/g)	K (g/kg)
R	37.47	43.07	19.07	2.63	1.4	7.76
RC	34.04	51.71	02.51	2.57	4.92	3.44
TF	36.27	51.49	06.99	2.58	1.16	4.71
TO	38.2	46.2	17.92	3.13	1.51	7.7
MB	30.48	55.41	05.98	2.58	2.09	3.93
MC	38.24	43.62	09.07	3.12	3.01	7.76
FP	34.66	54.75	07.18	2.87	4.42	7.86
FB	36.54	53.47	07.78	2.58	1.35	2.87
FL	34.75	50.28	05.77	2.57	1.12	6.7
